# Overexpression of Hypoxia-Inducible Factor-1α and Its Relation with Aggressiveness and Grade of Oral Squamous Cell Carcinoma

**DOI:** 10.3390/diagnostics13030451

**Published:** 2023-01-26

**Authors:** Sumera Sumera, Asif Ali, Yasar M. Yousafzai, Zubair Durrani, Mohammed Alorini, Benish Aleem, Rabia Zahir

**Affiliations:** 1 Institute of Pathology and Diagnostic Medicine, Khyber Medical University, Peshawar 25100, Pakistan; 2 College of Medicine, Gulf Medical University, Ajman P.O. Box. 4184, United Arab Emirates; 3 Department of Oral and Maxillofacial Surgery, Rehman Medical Institute, Peshawar 25000, Pakistan; 4 Department of Basic Medical Sciences, Unaizah College of Medicine and Medical Sciences, Qassim University, Unaizah 56219, Saudi Arabia

**Keywords:** oral cancer, biomarkers, immunohistochemistry, prognosis, diagnosis

## Abstract

Hypoxia-inducible factor-1α (HIF-1α) has been shown to be involved in cancer metastasis in several cancer types. There is however conflicting evidence of HIF-1α expression with oral cancer prognosis. Therefore, this study set out to investigate HIF-1α overexpression and its relationship with the aggressiveness and grade of oral squamous cell carcinoma (OSCC) and to explore the diagnostic potential of HIF-1α overexpression in OSCC in a cohort of Pakistani patients. Immunostaining of HIF-1α was performed on 54 OSCC and 14 normal oral mucosa (NOM) tissue samples and various cut-offs were used to evaluate its immunohistochemical expression. HIF-1α expression in OSCC samples was significantly higher than in controls, with minimal immunoreactivity in NOM. HIF-1α overexpression was significantly associated with increased tumor size (*p* = 0.046). However, no association was found between HIF-1α overexpression and increasing Broder’s histological grade or TNM stage. The cut-off >10% cells with moderate to marked intensity carried a sensitivity of 70% and a specificity of 100% to distinguish between tumor and control. ROC curve analysis of HIF-1α weighted histoscores showedHIF-1α overexpression as a highly sensitive and specific diagnostic test (*p* < 0.001, AUC = 0.833). HIF-1α overexpression is a tumor-specific finding associated with increased tumor size and carries a potential diagnostic role.

## 1. Introduction

Oral cavity cancer as the sixth most prevalent cancer in the world is a serious and growing problem. In South Asian high-risk countries, including Pakistan, India, and Bangladesh, it accounts for up to 25% of new cancer cases. About 90% of these cancers are oral squamous cell carcinomas (OSCCs). OSCC originates within a field of precancerized epithelial tissue, which could either be because of a pre-existing potentially malignant lesion or could be de novo. Despite the advancements in diagnostic and therapeutic strategies, the mortality rates remain unchanged for advanced-stage OSCCs with a five-year survival rate of only 40% [1,2,3]. The most important prognosticator in these patients is the TNM stage. Advanced TNM stage is shown to be associated with poor treatment outcome and vice versa. However, several early-stage OSCC patients still relapse, whereas some late-stage patients show good treatment responses. There is a need to identify more sensitive predictors of tumor relapse and aggressive behavior for better risk stratification [2,4].

Hypoxia-inducible factor-1α (HIF-1α) is one key transcriptional factor released in response to hypoxia within a rapidly growing tumor. In the presence of normal cellular oxygen levels, HIF-1α is quickly degraded by binding with the Von Hippel–Lindau (VHL) protein. However, in hypoxic conditions, the hydroxylation and subsequent destruction of HIF-1α does not take place, thus allowing its accumulation in these hypoxic cells. Stabilized HIF-1α binds to the hypoxia-responsive elements (HREs), resulting in the expression of more than 70 hypoxia-response proteins. These are hypothesized to be involved in changes in the metabolism, adhesion, and invasiveness of tumor cells [5]. Several clinical studies have shown HIF-1α overexpression is related to poor prognoses in cervical, breast, ovary, endometrium, gastric, and head and neck cancers [6,7,8,9,10,11]. However, some authors reported controversial results in head and neck SCCs, either showing no influence of HIF-1α overexpression on patient prognosis or its relationship with an improved survival rate [6,7,12].

While there is clear evidence of involvement of an HIF-1α-dependent mechanism in cellular metabolism and consequent survival, proliferation, and invasion, this has not been translated as a clinically reliable biomarker in OSCC. One of the reasons for this may lie in the reproducibility of HIF-1α IHC staining and the cut-offs at which a sample is considered over- or under-expressed for HIF-1α expression. We determined the expression of HIF-1α in OSCC tissue samples from a Pakistani population and assessed its correlation with the clinicopathological features. We analyzed this biomarker’s potential diagnostic role specifically in OSCC using various histological cut-offs.

## 2. Materials and Methods

### 2.1. Study Setting

This retrospective cohort study was carried out at the Institute of Basic Medical Sciences (IBMS), Khyber Medical University (KMU) in the year 2017. Prior approval was obtained from the Advanced Studies and Research Board of Khyber Medical University (KMU), Peshawar and the research committees of Rehman Medical Institute (RMI) and Khyber College of Dentistry (KCD), Peshawar.

### 2.2. Patient Selection

Sample size was calculated using OpenEpi software (version 3.01, OpenEpi Collection of Epidemiologic Calculators). Assuming proportions of exposed and unexposed for the outcome of 55% and 9%, respectively, a sample size of 41 (27 oral SCC and 14 normal oral mucosa) is sufficient to provide 80% power to the study. However, we included 13 more OSCC specimens (*n* = 54), and thus, the total sample size was 68.

Patients were identified using RMI hospital management information system (HMIC). OSCC patients of all age groups and both genders were included in this study. Patients who underwent chemotherapy or radiotherapy prior to the tumor excision were excluded from our study. Other clinicopathological data collected from the hospital medical records included age, gender, clinical TNM staging, tumor location, tumor size, histological grade, and the number of lymph nodes involved. A total of 54 OSCC formalin-fixed, paraffin-embedded (FFPE) tissue blocks were retrieved from the pathology department of RMI.

Normal oral mucosa (NOM) control samples included in this study were taken from healthy individuals undergoing elective extraction of their completely impacted third molars where reflection of oral mucosa was required. Tissues of 3 mm diameter were surgically excised, and FFPE tissue blocks were prepared using the standard histopathology protocol. The NOM tissues were devoid of any inflammation or pathology. Prior history and consent were taken from these individuals and tobacco users were excluded.

### 2.3. Histology and Immunohistchemistry

IHC was performed for the detection of HIF-1α in the OSCC and the NOM tissue samples. Two consecutive histological sections of 4µm thickness were taken from both the OSCC and NOM FFPE tissue specimens for hematoxylin and eosin (H&E) and IHC staining [13].

**Figure 1:**
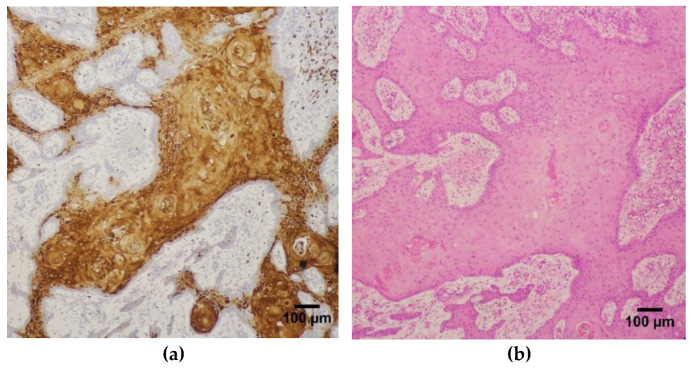
Photomicrographs of consecutive sections of SCC of the buccal mucosa showing sheets of tumor cell population. (**a**) IHC for HIF-1-α immunopositivity with minimal background staining using the final IHC parameters derived from the antibody optimization protocol (magnification ×100). (**b**) Corresponding Hematoxylin and Eosin stained section. 100× magnification, scale bar represents 100 micrometer.

For IHC, antibody optimization was carried out before the final staining of both the OSCC and NOM tissue sections to achieve the best results with minimal background staining. Tissue sections from an advanced stage metastatic OSCC tumor sample were used as a positive control, whereas for the negative control, the primary antibody was replaced with phosphate-buffered saline (PBS). IHC parameters employed for the final immunostaining of our OSCC samples and the resultant HIF-1α immunostaining are shown in Table 1 and Figure 1. Antigen retrieval was carried out using heat-induced epitope retrieval (HIER) where sections were immersed in pre-heated target retrieval solution (Tris/EDTA buffer, pH 9—EnVision FLEX detection kit, cat. no. K802321-2, Dako, Agilent Technologies, Santa Clara, CA, USA) and incubated in a hot air oven for 30 min at 100 °C. The sections were incubated for 15 min in peroxidase-blocking agent (EnVision FLEX detection kit, cat. no. K802321-2, Dako, Agilent Technologies, Santa Clara, CA, USA) to block the endogenous peroxidase activity [14]. Sections were then incubated with a rabbit monoclonal HIF-1α antibody (1:500; clone EP1215Y, Abcam, Cambridge, UK) at room temperature for 40 min in a humidity chamber. Secondary antibody (Dako, Agilent Technologies, Santa Clara, CA, USA) was applied next, and the slides were incubated in a humidity chamber at room temperature for 40 min. The antibodies were visualized using freshly prepared 3,3’ Diaminobenzidine tetrahydrochloride chromogen (Cat. no. K802321-2, Dako, Agilent Technologies, Santa Clara, CA, USA). Sections were counterstained using hematoxylin (EnVision FLEX Hematoxylin, cat. no. K8008, Dako, Agilent Technologies, Santa Clara, CA, USA) and dehydrated in alcohol and xylene before they were mounted [14].

### 2.4. Scoring

The H&E slides of OSCC tissue samples were histologically graded according to Broder’s grading system as well as Anneroth’s grading system. Broder’s histologic grading system is a well-established criteria for histological grading of tumors based on the degree of differentiation of the tumor cell population [15]. Anneroth’s grading system on the other hand not only takes into account the features of tumor cell population but also the features of the tumor–host relationship, such as pattern of invasion, stage of invasion, and lymphoplasmacytic infiltration; each parameter is scored from 1 to 4 points, and the sum of all scores is grouped as: 6–12 grade I, 13–18 grade II, 19–24 grade III [16].

**Figure 2:**
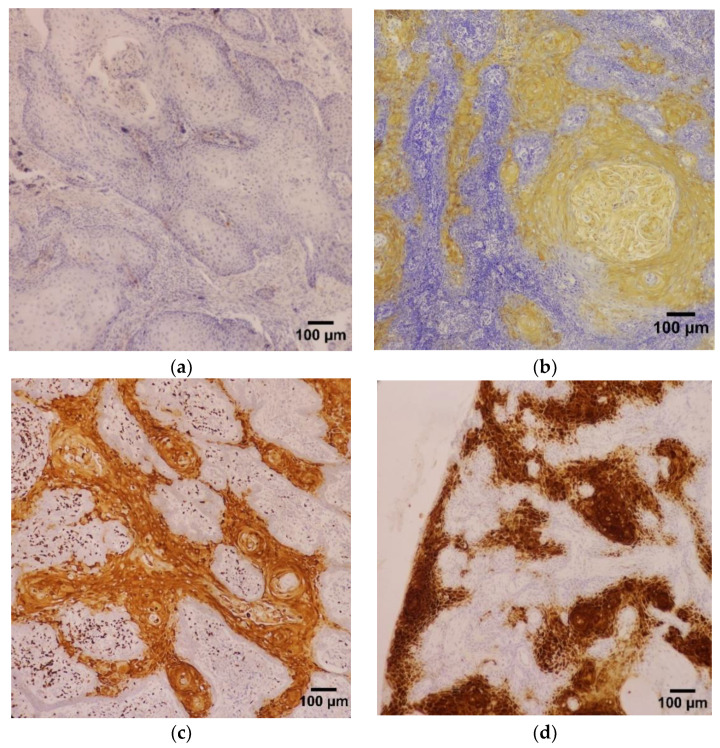
Photomicrographs of immunohistochemical staining of HIF-1-α in OSCC samples and the various staining intensities (magnification ×100): (**a**) staining intensity 0 (none), (**b**) staining intensity 1 (weak), (**c**) staining intensity 2 (moderate), (**d**) staining intensity 3 (strong).

**Figure 3:**
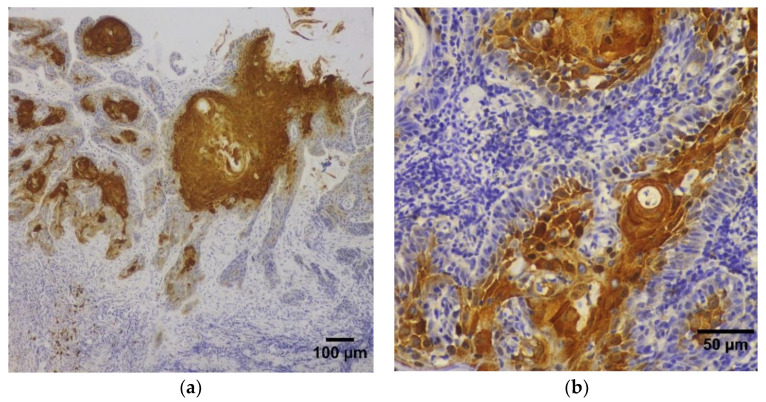
Photomicrographs of immunohistochemical staining for HIF-1-α in OSCC tumor samples: (**a**) SCC of the oral tongue infiltrating the underlying stroma with tumor islands exhibiting central areas of intensive HIF-1-α immunostaining (magnification ×100), (**b**) SCC of the oral tongue showing tumor islands with central zones of HIF-1-α immunoreactivity in the nuclear and cytoplasmic compartments of the tumor cells (magnification ×400).

The immunohistochemically stained tumor sections were viewed under a light microscope (Olympus CX21LED) and scored at a magnification of ×100. The observer was blinded to all the clinicopathologic parameters. The four methods used for IHC scoring included the immunoreactive score (IRS), weighted histoscore (HS), percentage of positive cells, and lastly, the proportion of positively stained cells and their intensity (Table 2 and Figure 2) [17,18,19,20]. Tumor cells were considered positive for HIF-1α immunoreactivity when there was nuclear staining with or without cytoplasmic staining (Figure 3). For calculating IRS and the weighted HS, five random separate fields were chosen for each stained section, and their IRS and HS were determined. The overall score for each slide was the mean of the values of the five separate fields. The scores were used to categorize the samples as HIF-1α ‘under-expression’ or ‘overexpression’.

### 2.5. Statistical Analysis

The relationships between HIF-1α expression categories (assessed using all the four cut-offs) and the various clinicopathologic parameters of tumor samples were evaluated using the chi-squared test (χ^2^ test). The diagnostic accuracy and ability of HIF-1α positivity to distinguish between diseased OSCC and NOM cases was determined by calculating the sensitivity and specificity for all the four cut-offs used for HIF-1α positivity. Furthermore, the receiver operating characteristic (ROC) curve analysis was also performed to assess the diagnostic accuracy of HIF-1α weighted HS values in both OSCC and NOM samples. SPSS Version 23.0 was used for statistical analysis. *p* values <0.05 were considered significant.

## 3. Results

### 3.1. Baseline Characteristics of the Study Population

The study consisted of OSCC specimens from 54 patients, among which 29 (53.7%) were male and 25 (46.3%) female with a median age of 60 years (SD± 14.64). The most commonly affected site was the tongue (twenty-one cases, 38.9%), followed by the buccal mucosa (nineteen cases, 35.2%), alveolar ridge (seven cases, 13%), lip (four cases, 7.4%), floor of the mouth (two cases, 3.7%), and lastly, the hard palate (one case, 1.9%). Out of fourteen NOM samples, nine were male and five were female. The median age of the healthy control population was 25.3 years (SD ± 5.6).

As per Broder’s histological grading of the fifty-four OSCC cases, thirty-seven (68.5%) were well differentiated (Grade 1), twelve (22.2%) were moderately differentiated (Grade 2), and five (9%) were poorly differentiated (Grade 3). Whereas, according to Anneroth’s histological criteria, 23 (42.5%) cases were Grade 1, and 31 (57.4%) were Grade 2. TNM stage I was the most frequent (n = 21), followed by stage III (n = 14), stage II (n = 10), and stage IV (n = 9) (Table 3).

### 3.2. HIF-1α Is Over-Expressed in OSCC

The comparison of HIF-1α expression in OSCC and NOM showed over-expression of HIF-1α staining in the majority of OSCC samples, whereas most NOM samples showed under-expression. Four different scoring systems were used for the quantification of HIF-1α staining (methods Section 2.4 and Table 2). Over-expression of HIF-1α was seen in 30/54 (55.6%) samples as per the weighted histoscore criteria, 37/54 (68.5%) as per ‘% positive cells 50%’ criteria, 38/54 (70.4%) as per ‘% positive cells and their intensity >10%’ criteria, and 42/54 (77.8%) as per Remmele IRS criteria. Remarkably, none of the NOM samples showed over-expression in the first three criteria, and only 4/14 (28.5%) NOM samples showed over-expression as per Remmele IRS criteria. This difference was highly statistically significant for each criteria (Table 2 and Table 4).

### 3.3. Association between HIF-1α Expression and Clinicopathological Features

Correlations between HIF-1α expression levels and clinicopathologic variables (age, gender, Broder’s histological grade, Anneroth’s histological grade, cTNM stage, tumor size, and nodal involvement) were assessed. No significant association was noted between HIF-1α expression levels assessed using all four cut-offs and the clinicopathological parameters, namely age, gender, cTNM stage, nodal involvement, tumor site, and Broder’s histological grade. A statistically significant association was found between HIF-1α overexpression and increased tumor size (T status) using the weighted histoscore (median value HS120) as a cut-off for positivity (*p* = 0.046; χ²-test). However, the remaining HIF-1α quantifications (cut-offs) did not show this positive association. An inverse association was noted between HIF-1α overexpression and increasing Anneroth’s histologic grade using two HIF-1α cut-offs (HS120 *p* = 0.019, percent positive cells and cut-off >50% *p* = 0.012).

### 3.4. Diagnostic Value of HIF-1α Expression in OSCC

**Figure 4:**
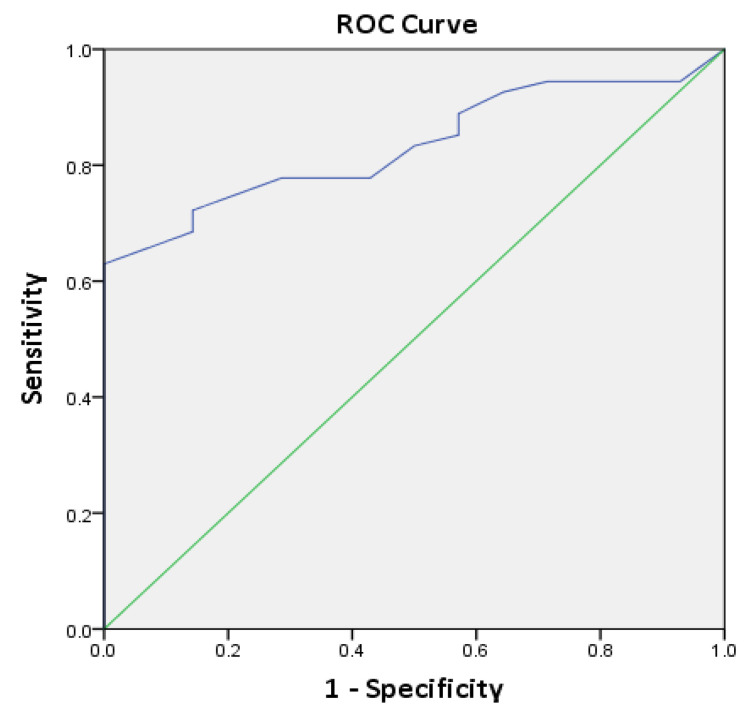
ROC curve based on HIF-1α weighted histoscores (HS) in NOM and OSCC samples. AUC = 0.833 (95% CI, *p* < 0.001).

Since HIF-1α expression in normal tissues is low to none, we sought to evaluate HIF-1α overexpression as a diagnostic biomarker assessing its sensitivity and specificity using all four cut-offs (quantifications). The cut-off carrying the highest diagnostic accuracy was >10% cells with moderate-to-marked intensity (sensitivity = 70%, specificity = 100%). The diagnostic accuracy of HIF-1α increased expression calculated using weighted HS was also assessed through ROC curve analysis. The area under the curve (AUC) for HIF-1α was 0.833 (95% CI, *p* < 0.001) (Figure 4).

## 4. Discussion

HIF-1α is known to be overexpressed in a number of cancer types [6,7,8,9,10,11]. However, its use as a diagnostic and prognostic marker has not been established. This study, for the first time, assessed HIF-1α expression in a Pakistani cohort of OSCC patients. We showed increased expression of HIF-1α is a tumor-specific finding. The results showed a substantial increase in HIF-1α protein expression in OSCC samples in comparison to NOM samples. Using HS120 as a cut-off, 30 out of 54 OSCCs showed HIF-1α overexpression. A statistically significant association between HIF-1α overexpression and increased tumor size was also noted using the same cut-off for positivity (HS120). No statistically significant association could be established between HIF-1α overexpression and other clinicopathologic variables, including clinical TNM stage, nodal status, tumor site, age, gender, and histological differentiation, i.e., Broder’s grade. HIF-1α expression achieved 70% sensitivity and 100% specificity in discriminating between OSCC and NOM samples.

Despite that HIF-1α overexpression in OSCC is now considered an established fact, its use as a clinically useful marker has been limited by the conflicting results of prognostic studies [6,7,12]. Several studies have demonstrated the prognostic significance of HIF-1α overexpression in OSCC tumors, owing to its association with an advanced TNM stage, nodal involvement, increased tumor size, tumor recurrence, and poor survival [6,7,8,9,10,11,21]. This interpretation is supported by the cellular mechanisms of HIF-1α function. Enhanced activation of the HIF-1α system in sustained hypoxia conditions within tumors leads to certain cellular changes that promote tumor progression. The result is a clinically aggressive tumor phenotype with a propensity for locally invasive growth, regional and distant metastasis, and a worse prognosis [22]. The positive association observed between HIF-1α overexpression and an increased tumor size using HS120 as a cut-off for positivity in our study indicates increased activation of the HIF-1 system in these tumors certainly resulted in an increased potential for growth. Although HIF-1α overexpression was found to be significantly associated with tumor size, associations with other clinicopathologic parameters were not noted. These findings are in agreement with those of Eckert et al. [17].

The lack of association with clinicopathological parameters except tumor size could be the result of assessing HIF-1α staining in a single section from a biopsy, which can provide only a snapshot of the whole dynamic hypoxia environment within the tumor [23]. The possibility of surgery-induced ischemia altering HIF-1α expression in the subsequent biopsies and at times the extended fixation times used for these tissues may further hinder the ability to establish the exact HIF-1α protein expression levels in these tumors [22]. Despite these methodological challenges in calculating HIF-1α protein expression levels via immunohistochemistry, the positive association found between tumor size and HIF-1α overexpression in our study shows increased HIF-1α signaling in OSCC is indeed related to tumor growth, progression, and a larger tumor size (advanced T status), thus proving the prognostic significance of this biomarker.

Broder’s histological grade of tumors failed to show any association with HIF-1α overexpression upon statistical analysis. Anneroth’s histological grade however did show an association with HIF-1α overexpression, and interestingly, this relationship was found to be of an inverse nature. The observed inverse association is in agreement with Aebersold et al., but the opted histologic grading system was Broder’s, not Anneroth’s [20]. Throughout the literature, there have been confusing answers as to whether HIF-1α overexpression is related to an increasing histological grade or not. Some authors (Eckert et al. and Yamada et al.) have reported no such association, whereas others (Kang et al.) have reported a positive association, and then there are some (Aebersold et al.) who have reported an inverse association between the two [20,24,25,26]. If this relationship between HIF-1α overexpression and histological grade of OSCC exists, its nature is yet to be defined because the results so far seem to be controversial and need further clarification.

As a diagnostic biomarker, HIF-1α overexpression was found to have a high sensitivity and specificity when used with >10% cells with moderate-to-marked intensity as a cut-off. ROC curve analysis of HIF-1α expression weighted histoscores (HS) in the OSCC samples and NOM samples showed a large AUC, proving the diagnostic accuracy of HIF-1α overexpression in OSCC. Using the same analysis, a list of potential histoscore cut-off values was also generated, each having specific sensitivity and specificity values. Further validation studies are needed for the clinical translation of HIF-1α as a diagnostic biomarker, but so far, we agree with Sayáns et al. who believe HIF-1α to be a suitable marker for diagnostic purposes in OSCC [3,6].

The diagnostic accuracy of HIF-1α overexpression noted in our study has led us to believe the inclusion of assessment of HIF-1α overexpression in OSCC biopsies at the time of diagnosis may not only help in diagnosing the ongoing malignant OSCC process but can also help in marking OSCC tumors with a clinically aggressive phenotype with a propensity for unrestricted growth. This could help in OSCC patient stratification and personalize cancer treatment because the HIF-1α pathway is a potentially treatable and‘druggable’ target. Several direct and indirect inhibitors of HIF-1α are already in preclinical and clinical development, which can limit HIF-1α related tumor growth, angiogenesis, and tumor progression in many solid human tumors, including OSCC [3,27].

To accurately characterize the extent of hypoxia levels within a tumor, a panel of endogenous hypoxia markers should be used instead of a single biomarker. This panel of hypoxia markers, besides HIF-1α, should include its main downstream proteins, such as the glucose transporter 1 (GLUT-1), carbonic anhydrase IX (CAIX), and vascular endothelial growth factor (VEGF) [28]. Therefore, we suggest more large-scale studies in our population to assess the prognostic and diagnostic significance of HIF-1α overexpression and its co-expression with other main downstream proteins as a part of a panel of hypoxia biomarkers in OSCC.

Despite showing scientifically valid observations, in a previously unexplored Pakistani population, the study is not without limitation. There was limited clinical data and treatment and survival data could not be collected, hindering the survival analysis necessary for establishing its prognostic role. The other limitation is a relatively small sample size. HIF-1α expression levels at the transcript level were not determined as well. However, the limited inferences we draw from these analyses appear scientifically rational and are consistent with published reports from elsewhere. As to why a prognostic role of HIF-1α cannot be identified remains to be concluded and will probably be answered in a much larger longitudinal cohort or mechanistic laboratory studies.

## 5. Conclusions

It could be concluded from this study that increased expression of HIF-1α is a tumor-specific finding, with little to no expression in normal oral tissues. Assessment of HIF-1α overexpression in OSCCs can improve diagnostic accuracy. The association of HIF-1α expression with clinicopathologic features and disease course needs further studies.

## Figures and Tables

**Table 1:** Immunohistochemistry-staining protocol.

Primary Antibody	Primary Antibody Dilution	HIER Method	Antigen Retrieval Time	Antigen Retrieval Temp.	Peroxidase Blocking	Primary Antibody Incubation	HRP Incubation
Rabbit monoclonal HIF-1α antibody, clone EP1215Y, Abcam	1:500	Hot air oven	30 min	100 °C Tris EDTA pH = 9.0	15 min	40 min	40 min

**Table 2:** The four quantifications and their set cut-offs used for HIF-1α scoring and data analysis.

Quantifications and Their Cut-Offs	Under-Expression	Overexpression
Weighted Histoscore (HS 0-300): [18] Cut-off used ≥120 (median HS value)	<120	≥120
Percent positive cells: [19] (1+) 1–10% (2+) 11–50% (3+) 51–80% (4+) 81–100% Cut-off used >50%	≤50%	>50%
Percent positive cells and their intensity: [20] + = <1% ++ = 1–10% slight to moderate +++ = 10–50% moderate to marked ++++ = >50% moderate to marked Cut-off used >10%cells with moderate to marked intensity	≤10%	>10%
Remmele IRS: [17] IRS 0–-1 = no staining IRS 2–3 = weak staining IRS 4–8 = moderate staining IRS 9–12 = strong staining Cut-off used IRS ≥4	<4	≥4

**Table 3:** Clinicopathologic parameters of OSCC patients.

Clinicopathologic Parameters of OSCC Patients		Observation (n = 54)
Age	<60 ≥60	25 (46.3%) 29 (53.7%)
Gender	Male Female	29 (53.7%) 25 (46.3%)
Site distribution	Oral tongue	21 (38.9%)
	Buccal mucosa	19 (35.2%)
	Alveolar ridge	7 (13%)
	Lip	4 (7.4%)
	Floor of the mouth	2 (3.7%)
	Hard palate	1 (1.9%)
Broder’s histologic grade	G1 G2 + G3	37 (68.5%) 17 (31.4%)
Anneroth’s histologic grade	G1 G2	23 (42.5%) 31 (57.5%)
TNM stage	Stage I + II Stage III + IV	35 (64.8%) 19 (35.1%)

**Table 4:** Clinical, histopathological, and immunohistochemical data of OSCC specimens and their associations with HIF-1α expression.

Clinicopathological Variables	n (%)	The Four Quantifications and Their Cut-Offs Used to Assess HIF-1α Immunoreactivity							
		Weighted Histoscore:		% Positive Cells:		% Positive Cells and Their Intensity:		Remmele IRS:	
		*Cut-off ≥120 (HS120)*		*Cut-off >50%*		*Cut-off >10%*		*Cut-off IRS ≥4*	
		Under-Expression	Over-Expression	Under-Expression	Over-Expression	Under-Expression	Over-Expression	Under-Expression	Over-Expression
**Tissue samples**		***p*** **-value 0.00**		***p*** **-value 0.00**		***p*** **-value 0.00**		***p*** **-value 0.00**	
NOM	14	14	0	14	0	14	0	10	4
OSCC	54	24	30	17	37	16	38	12	42
**Gender**		*p*-value 0.30		*p*-value 0.21		*p*-value 0.12		*p*-value 0.34	
Male	29 (53.7%)	11	18	7	22	6	23	5	24
Female	25 (46.3%)	13	12	10	15	10	15	7	18
**Age (years)**		*p*-value 0.30		*p*-value 0.06		*p*-value 0.12		*p*-value 0.34	
<60	25 (46.3%)	13	12	11	14	10	15	7	18
≥60	29 (53.7%)	11	18	6	23	6	23	5	24
**Tumor site**		*p*-value 0.93		*p*-value 0.58		*p*-value 0.58		*p*-value 0.66	
Tongue	21 (38.9%)	10	11	8	13	7	14	5	16
Buccal mucosa	19 (35.2%)	8	11	6	13	4	15	3	16
Other sites	14 (25.9%)	6	8	3	11	5	19	4	10
**Broder’s grade**		*p*-value 0.14		*p*-value 0.09		*p*-value 0.53		*p*-value 0.38	
G1	37 (68.5%)	14	23	9	28	10	27	7	30
G2 + G3	17 (31.4%)	10	7	8	9	6	11	5	12
**Anneroth’s grade**		***p*** **-value 0.01**		***p*** **-value 0.01**		*p*-value 0.09		*p*-value 0.16	
G1	23 (42.5%)	6	17	3	20	4	19	3	20
G2	31 (57.5%)	18	13	14	17	12	19	9	22
**Nodal status**		*P*-value 0.43		*p*-value 0.53		*p*-value 0.91		*p*-value 0.49	
N0	41 (75.9%)	17	24	12	29	12	29	10	31
N1 + N2 + N3	13 (24.0%)	7	6	5	8	4	9	2	11
**Tumor size**		***p*** **-value 0.046**		*p*-value 0.38		*p*-value 0.61		*p*-value 0.65	
T1	23 (42.5%)	7	16	6	17	6	17	5	18
T2	12 (22.2%)	9	3	6	6	5	7	4	8
T3	17 (31.4%)	8	9	4	13	5	12	3	14
T4	02 (3.70%)	0	2	1	1	0	2	0	2
**TNM stage**		*p*-value 0.85		*p*-value 0.74		*p*-value 0.50		*p*-value 0.40	
Stage I + II	35 (64.8%)	13	17	10	20	10	20	9	26
Stage III + IV	19 (35.1%)	11	13	7	17	6	18	3	16

## Data Availability

The data set used in the current study will be made available on request from Sumera Sumera, dr.sumera91@gmail.com.
